# Light‐Controlled Conductive Molecular Wire in an Artificial Lipid Membrane

**DOI:** 10.1002/advs.77303

**Published:** 2026-08-21

**Authors:** Ilaria Cardace, Lorenzo Dominici, Adriano Cola, Antonio Fieramosca, Vincenzo Ardizzone, Agostina Lina Capodilupo, Bruno Rizzuti, Milena De Giorgi, Dario Ballarini, Giuseppe Gigli, Daniele Sanvitto, Luisa De Marco

**Affiliations:** ^1^ CNR‐NANOTEC Institute of Nanotechnology Lecce (LE) Italy; ^2^ CNR‐IMM Institute for Microelectronics and Microsystems Lecce (LE) Italy; ^3^ CNR‐NANOTEC Institute of Nanotechnology Rende (CS) Italy; ^4^ Institute of Biocomputation and Physics of Complex Systems University of Zaragoza Zaragoza Spain; ^5^ Department of Experimental Medicine University of Salento Lecce Italy; ^6^ Tecnomed Puglia – Tecnopolo per la medicina di precisione (Biotech Lecce Hub) c/o Campus Ecotekne Lecce Italy

**Keywords:** artificial lipid membrane, azobenzene derivatives, bio‐electronics, molecular wire, optoelectronic bio‐interfaces, photoswitching molecules

## Abstract

In this work, we investigate a novel photochromic azobenzene derivative, **TC827**, which exhibits a unique photoelectric response when embedded in artificial lipid membranes. By combining spectroscopic, electrochemical, and molecular simulation analyses, we demonstrate that **TC827** forms light‐controlled “molecular wires” in its trans conformation, resulting in a significant increase in membrane conductivity under dark conditions, which is fully reversible, as light‐induced cis isomerization suppresses the conductivity enhancement. This behavior is fundamentally different from previously studied amphiphilic azobenzene derivatives, whose effect on lipid membranes is usually attributed to their ability to increase permeability by disrupting membrane packing, leading to a current increase in the cis state under irradiation. In our system, the increase in conductivity is not associated with membrane destabilization due to the chemical structure of **TC827** molecule, which lacks long alkyl chains and features a π‐conjugated structure. These findings underline the potential of structurally simple, short‐chain photo‐switchable molecules to modulate membrane properties through charge transport mechanisms induced by light, offering a new pathway toward the design of optoelectronic bio‐interfaces.

## Graphical abstract

A photochromic azobenzene derivative is shown to produce a unique photoelectric response when embedded in artificial lipid membranes. In its trans state, it forms conductive “molecular wires” that enhance membrane conductivity in the dark. Light‐induced cis isomerization suppresses this effect, showing that short‐chain molecules can modulate membrane properties and enable reversible light‐controlled optoelectronic biointerfaces.

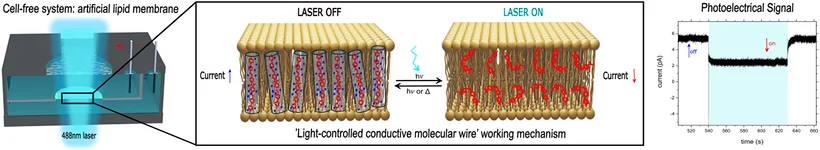

## 1 Introduction

In recent years, the intersection of biology and electronics is leading to the development of bioinspired technologies that could result in flexible, biodegradable, and biocompatible devices. Biological systems, refined by nature over millions of years of evolution, offer structures and functions that are more complex and efficient than many synthetic materials. Recently, these natural architectures have been utilized to create optoelectronic devices with improved performance, better sustainability, and new functionalities [1, 2, 3, 4, 5, 6].

One of the most promising areas of this interdisciplinary research is using biological structures like proteins and membranes as active components in photonic devices, offering benefits such as self‐assembly and nanoscale optical responsiveness, which are challenging to achieve through traditional fabrication methods [7].

In this field, photochromic molecules enable the modulation of the response to optical stimuli in a reversible manner [8, 9]. The most common examples of this family of compounds are derivatives of the azobenzene molecule that, upon exposure to light, interconvert between an extended, thermally relaxed trans isomer and a shorter, higher‐energy cis or “folded” isomer [10, 11].

Due to their ability to undergo multiple photoisomerization cycles without degradation or loss of reactivity [12], these molecules have been used as photoresponsive elements in membranes, ion channels, and cells [11, 13]. For example, azobenzene derivatives have been reported to optically control the activity of the human serotonin transporter [8, 14].

Furthermore, they enable precise light‐controlled modulation of membrane properties [15] such as its organisation, fluidity, permeability, and electrical response through nanoscale movements caused by their conformational changes [16, 17]. Consequently, they are often embedded into biological membranes as synthetic photo‐switchable lipid molecules, known as “photo‐lipids” [17, 18], opening new photopharmacological approaches for regulating inflammatory and immune and response [18, 19]. In most cases, meticulous molecular design followed by complex synthesis processes is required to ensure that these photochromic functionalities can be appropriately incorporated into the lipid membrane. For example, visible light pulses have been shown to trigger the firing of the action potential in neurons loaded with an engineered azobenzene‐based amphiphilic photoswitching molecule [20].

In the fields of bio‐electronics and bio‐photonics, acellular devices featuring artificial membranes, such as the so‐called Black Lipid Membrane (BLM) [21] are of great interest. This model system is a planar free‐standing bilayer of phospholipids formed between two compartments and offers several advantages compared to classical electrophysiological approaches in terms of sensitivity of electrical measurement, stability, and miniaturization. Acellular devices are ideal for understanding the working mechanism of photochromic materials in biological systems, isolating it from the complex phenomena occurring in living cells, where the presence of various membrane proteins and molecules can alter the membrane behavior. Therefore, this platform is the most suitable for isolating and studying the properties of a new molecule.

Yonezawa and co‐workers were among the first to demonstrate how photoisomerization can be used to modulate the electrical properties of artificial membranes in terms of permeability and conductivity. They observed that, upon UV irradiation, when the azobenzene molecules are in cis configuration, the membrane conductivity increases, likely due to a reduction in structural order and an increase in the permeability of the lipid bilayer. In these studies, as in many others, amphiphilic azobenzene derivatives with long organic chains, very similar to membrane lipids, were used [22, 23, 24, 25, 26].

In this work, rather than employing long‐chain azobenzene derivatives, we introduce a short and structurally simple dye, (E)‐4‐(4‐((4‐(diethylamino)phenyl)diazenyl)phenyl)‐1‐methylpyridin‐1‐ium iodide, hereafter referred to as molecule **TC827**, consisting of a methylpyridinium moiety directly linked to azobenzene functionality. We studied the light‐induced behavior of the lipid membrane embedding the **TC827** molecule in a microfluidic acellular device and, surprisingly, we observed a significant increase in conductivity in the dark, when the molecule is in its trans configuration, whereas light‐induced switching to the cis form suppresses this conductivity enhancement. This behavior is opposite to that normally observed for amphiphilic azobenzene derivatives, where only a minor increase in current is observed when the molecules are in cis configuration and therefore under light irradiation [23, 24, 25, 26].

By employing a combination of spectroscopic and electrical techniques, supported by molecular simulations, we demonstrate for the first time how a photo‐switchable molecule with a chemical structure significantly different from conventional “lipid‐like” molecules operates through a distinct and less disruptive mechanism for the membrane, which involves the formation of a “light‐controlled molecular wire.”

Our findings contribute to a deeper understanding of photo‐responsive membrane systems, adding new insights into the relationship between molecular structure and functionality in such devices. Furthermore, they can improve our understanding of how chemical structures can control the photo‐electrical properties of a membrane in a finely tuned manner.

## 2 Results

### 2.1 Synthesis and Optical Photoswitching Spectroscopy

**FIGURE 1:**
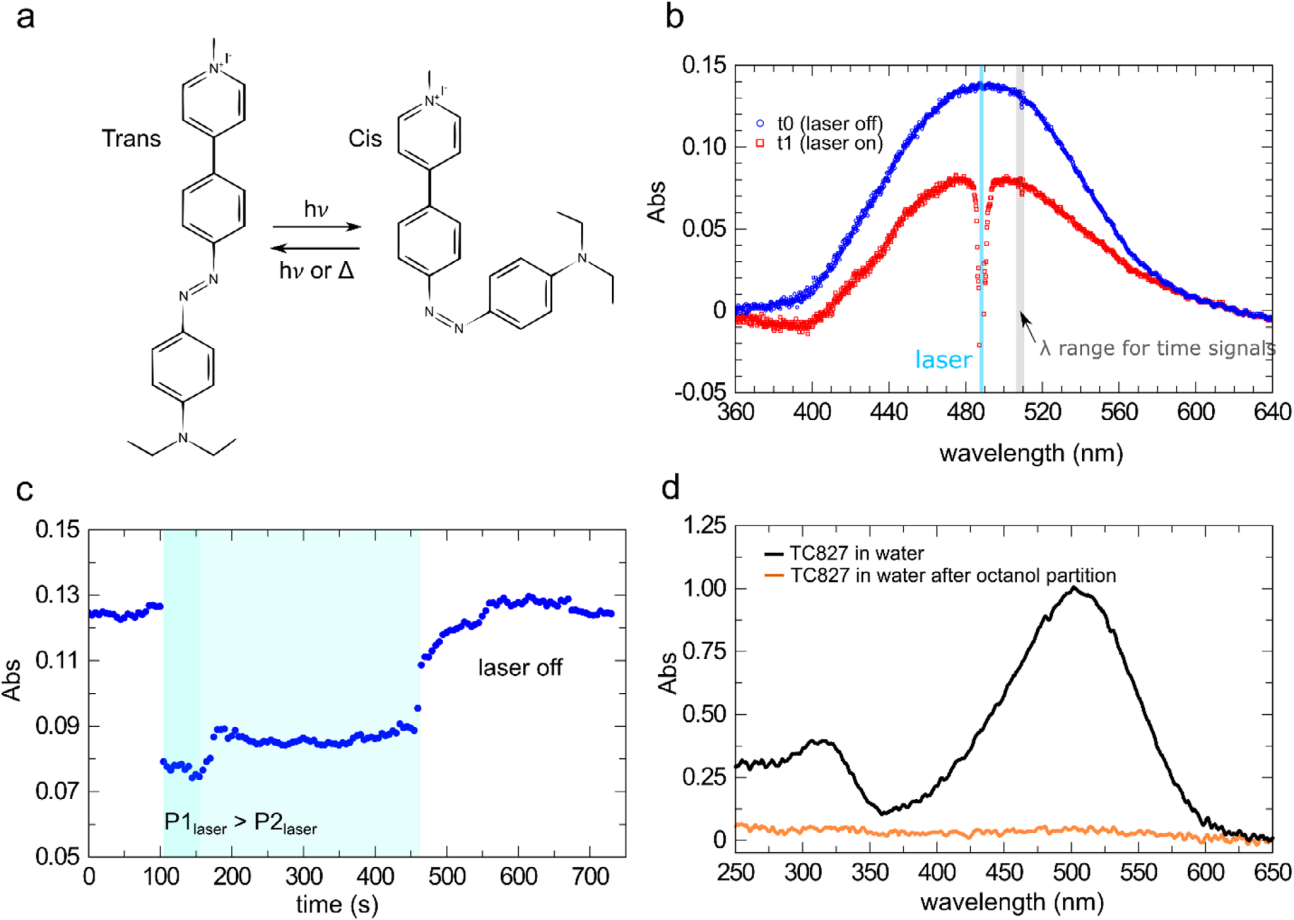
(a) Chemical structure of molecule **TC827** and representation of the trans‐cis photoisomerization reaction; the trans form possesses a quasi‐planar structure with a length of 17Å while the cis form shows a distorted structure with a length of 12Å. (b) Evolution of the absorbance spectrum for the **TC827** dye in a PMMA film before (blue trace) and during (red trace) laser excitation with 488nm wavelength. (c) Temporal dynamics of the absorption peak intensity (extracted at λ = 510 nm) during a 750 s time range (blue dots). The part from 100 to 450 s of the plot represents the irradiation with laser light at 488 nm (shaded cyan area), with a P_1_∼30 mW power from 100 s to 150 s and a P_2_∼15 mW power from 150 to 450 s, followed by the recovery interval from 450 to 750 s (laser off). (d) Absorption spectrum of molecule **TC827** in water solution before (black) and after (orange) octanol addition, emulsion, and recovery.

Compound **TC827** (Figure 1a) was synthesized following the route illustrated in the Supplementary Information. (E)‐4‐((4‐bromophenyl)diazenyl)‐N,N‐diethylaniline, **1**, was prepared by converting commercial 4‐bromoaniline to 4‐bromobenzenediazonium tetrafluoroborate with sodium nitrite and tetrafluoroboric acid at 0°C. The resulting diazonium salt was then reacted with diethylaniline. Under Suzuki reaction conditions, in a reaction mixture of toluene, water, and ethanol (2:1:1), 2 M sodium carbonate, and tetrakis(triphenylphosphine)palladium(0) as catalyst, compound **1** was reacted with pyridin‐4‐ylboronic acid by microwave irradiation to obtain compound **2**. Finally, compound **2** in the presence of an excess of methyl iodide in acetonitrile at room temperature, was transformed into compound **TC827**. Detailed synthetic protocols and product characterization data of all compounds are reported in Figures S1–S7.

As mentioned above, **TC827** is significantly different from other previously reported photoswitchable molecules used in biological or artificial membranes, because it does not have long alkyl chains. In contrast, it is relatively short, as the charged N‐methylpyridinium, the moiety that could interact with the polar heads of the lipids, is directly linked to the azobenzene. The linear geometry of the trans form could facilitate the intercalation of molecule **TC827** parallel to the phospholipids. Upon irradiation, the molecule switches to the cis form with bent geometry and increased lateral size (Figure 1a).

Before incorporating the **TC827** molecule into the artificial membrane, we first investigated its photoisomerization properties under direct laser irradiation. Specifically, we embedded the molecule in a polymeric matrix. We directly measured the temporal changes in the absorption spectrum by pumping near the trans‐state's absorption maximum (490nm). A broadband, low‐power white light source was used to monitor continuously the absorption spectrum while a strong cw pump laser (488nm) induced the trans‐cis molecular transition. Further details about the experimental setup can be found in the Methods section. Our approach is relatively simple and provides direct insight into the molecular modifications that occur upon laser irradiation.

The absorption spectra for the **TC827** molecule film before (blue trace) and during excitation (red trace) are shown in Figure 1b. As can be seen, the absorption is significantly reduced when the pump is active, while it approaches the initial value when the pump is turned off (see Figure 1c). This observation immediately suggests that the **TC827** molecule film undergoes photoisomerization. The temporal dynamics of the absorption peak intensity (extracted at λ = 510 nm) is plotted in Figure 1c as a function of time. For this data set, after an initial observation under dark (no laser) condition, the sample was exposed to the pump laser for ~50 s at P_1_ = 30 mW. The laser power was then reduced to P_2_ = 15 mW for ~300 s before being turned off. During the first laser exposure, a rapid step‐like absorbance reduction was observed, and a partial recovery of the absorbance was noted upon reducing the laser power from P_1_ to P_2_. The complete reversion to the initial condition occurred within 150 s after the laser was turned off. The spectral modifications observed, the step‐like temporal dynamics of the absorbance, and its complete reversion can be unambiguously interpreted as a cis‐trans photoisomerization process of the **TC827** molecule.

Since this molecule is relatively short and does not have long alkyl chains, we evaluated its phase partitioning between the lipid and water environment by calculating the n‐octanol/water partition coefficient (logP) to ensure that it could be properly embedded into the membrane. We observed that the initial concentration of the molecule in water, 17 mm, decreased to 0.8 mm after octanol addition, as shown in Figure 1d, demonstrating that the molecule has a much higher affinity for the hydrophobic environment and, therefore, its incorporation into the lipid bilayer should be favored.

Further partitioning experiments, which confirmed the molecule incorporation into the membrane, were also performed employing membrane‐like carriers, specifically nanodiscs formed by a lipidic bilayer surrounded by membrane scaffold protein with a size of 12 nm [27] (data are shown in Figure S8). A blue shift of 15 nm in the absorption peak was also observed (see Figure S8d) when the molecule is incorporated into the n‐octanol.

### 2.2 Photoelectric Measurements in an Acellular Device

**FIGURE 2:**
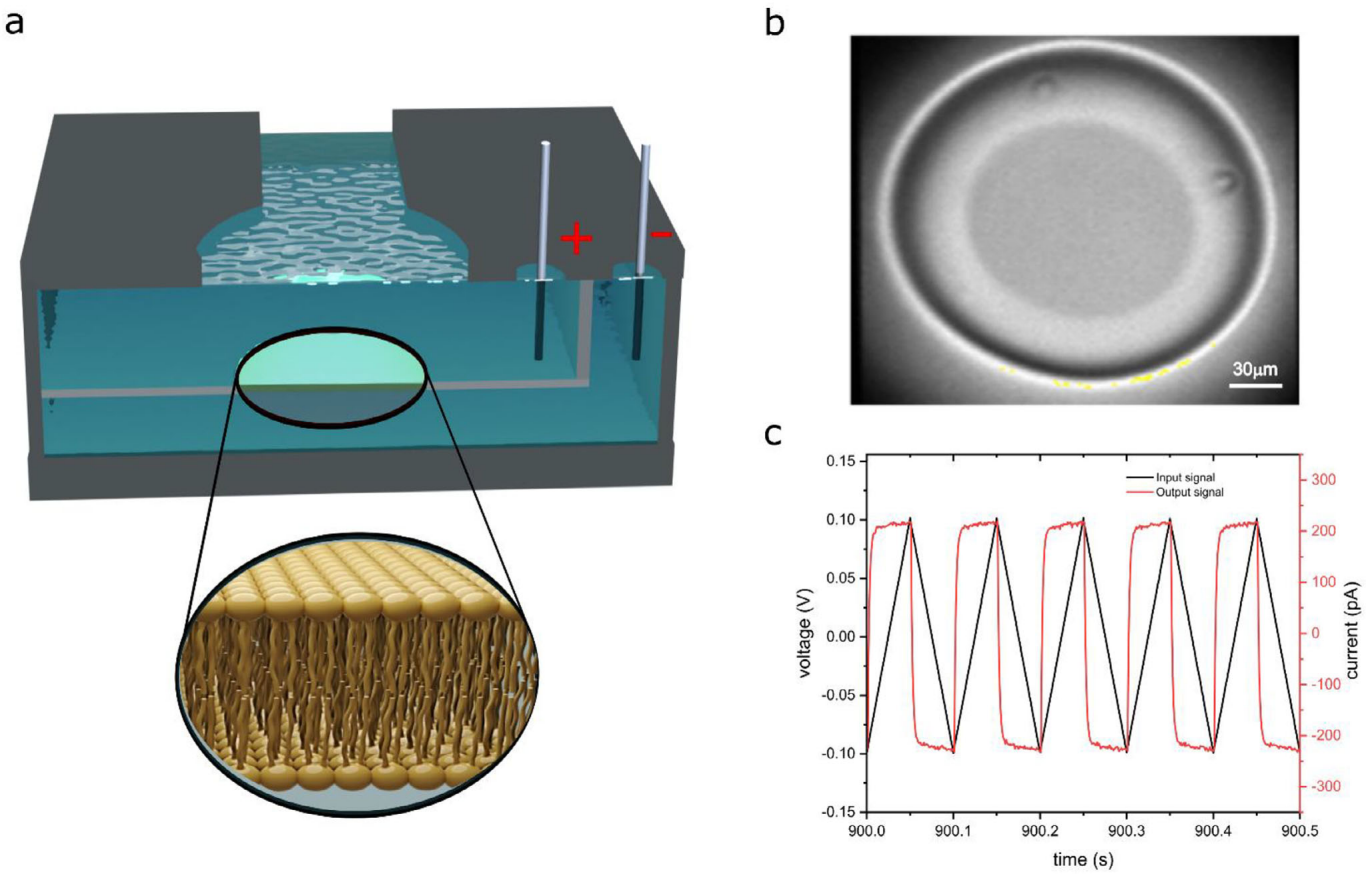
(a) Schematic representation of the microfluidic device. The lipid membrane is represented in the ∼100 mm hole on the Teflon septum between the two microchambers. These are filled with KCl 0.5 M, independently accessible and provided by electrodes. (b) Bright‐field image of a BLM formed across the septum using the air bubble technique with DPhPC in n‐octane (10 mg/mL) in the microfluidic device. (c) Typical current response of BLM upon application of a triangular voltage wave under dark, with the signal in input (black) and signal in output (red) showing a typical square wave.

To investigate the electrical properties of **TC827** on a lipid membrane, and particularly its behavior under optical irradiation, an acellular device was developed. A synthetic bilayer membrane, the so‐called BLM [21], was formed within a microfluidic device (Figure 2a) equipped with electrodes connected to a voltage generator and a current amplifier. The membrane was visualized using an optical microscope, with white light directed from the top while a lateral aperture in the microscope allowed the laser enters through the objective lens to irradiate the membrane. The microfluidic device consisted of two microchambers filled with an electrolyte solution (KCl 0.5 M) into which the two chlorinated silver electrodes were immersed. The two chambers were separated by a horizontal Teflon septum having a hole with a diameter of ∼100 µm in which the artificial membrane, composed of a diphytanoyl phosphatidylcholine (DPhPC) phospholipid, was formed utilizing the air bubble technique [28]. When lipids were added to the electrolyte solution from the upper microchamber in proximity to the hole, they initially arranged in thick and heterogeneous film that gradually thinned over time, primarily driven by van der Waals interactions between lipid molecules. To minimize their energy, the lipids self‐assembled with their polar head groups oriented outward toward the aqueous environment, while the hydrophobic tails aligned inward, forming a bilayer structure typical of biological membranes.

The formation of the membrane was electrically and optically monitored. From an optical point of view, the formation of the characteristic annulus can be observed (Figure 2b), the ring‐like region around the BLM where the lipids are in contact with the septum. The formation of the BLM is also verified by measuring the electric current between the chambers, upon application of an Alternate Current (AC) triangular voltage waveform (peak‐to‐peak amplitude 200 mV, frequency 10 Hz, black line in Figure 2c). In the absence of BLM, the path between the electrodes is purely resistive and the current waveform is triangular (not shown), a scaled replica of the input voltage. On the other hand, when the BLM is formed, since its lipid bilayer acts as an insulating dielectric, the output current becomes a square wave (Figure 2c, in red). The square wave stabilizes when the membrane reaches its final thickness, with the complete formation of the bilayer.

The associated capacitance C can be calculated by supposing a dominating capacitive term, as C = I/(dV/dt), where I is the measured current and dV/dt is the voltage slope in the time half‐period of the modulated waveform. The capacitance values measured in our device typically ranged from 20 to 50 pF in the presence of the BLM, depending on the diameter and thickness of the bilayer membrane. For instance, in the specific case shown in Figure 2c, the BLM had a diameter of 120 µm, a calculated capacitance of 50 pF and an estimated thickness of 4.4 nm, calculated using the capacitor model C = ε_0_εA/d (see Methods for further details). This value is in good agreement with typical lipid bilayer thicknesses of approximately 4–5 nm reported in the literature [29].

**FIGURE 3:**
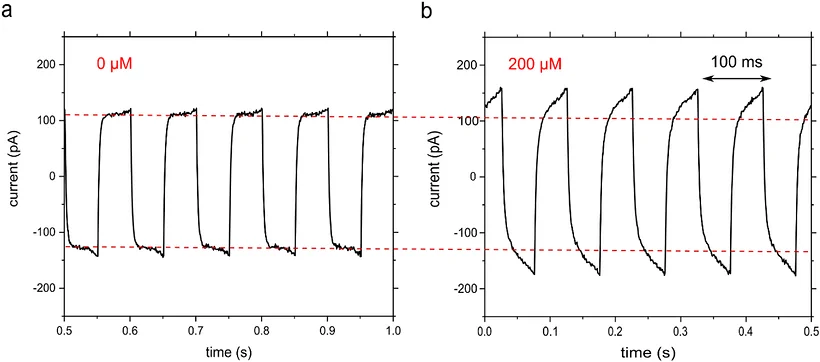
(a) Photoelectric measurement of the signal from the planar lipid bilayer upon application of an AC triangular wave voltage with 100 mV amplitude at 10 Hz modulation frequency, without any molecule addition in the dark, in a time range of 0.5 s. (b) The current response of the lipid membrane after the addition of the photoactive molecule **TC827** at a concentration of 200 µm in the dark upon applying the same AC triangular wave voltage, in a time range of 0.5 s.

We studied the AC waveform without and with the addition of the photoactive molecule to the membrane, as reported in Figure 3a,b, respectively.

The electrical response of the sole BLM had the typical square wave shape (Figure 3a). On the opposite, when molecule **TC827** was added (200 µm) to both the upper and lower chambers of the flowcell, we observed evident differences in the electrical traces (Figure 3b). A more detailed analysis of the output current signal waveform revealed changes in both the shape and the amplitude of the square wave, which goes from a square profile without **TC827** molecule (Figure 3a) to a more “stretched” shape after its addition (Figure 3b).

The increased slope of the current suggests a decrease in electrical resistance (i.e., an increase in conductance); simultaneously, the increase in the square wave amplitude indicates an enhancement in capacitance. These two effects are associated with the presence of **TC827** molecules in their trans configuration within the membrane in the dark [30]. The dependence of these changes on the concentration is shown in Figure S9a–f. Interestingly, the capacitance is slightly reduced when the molecules switch to their cis form under laser exposure, as shown in Figure S9.

We also examined the behavior of the photochromic molecule in independent direct current (DC) measurements by keeping the voltage constant at +100 mV and recording the current over time.

**FIGURE 4:**
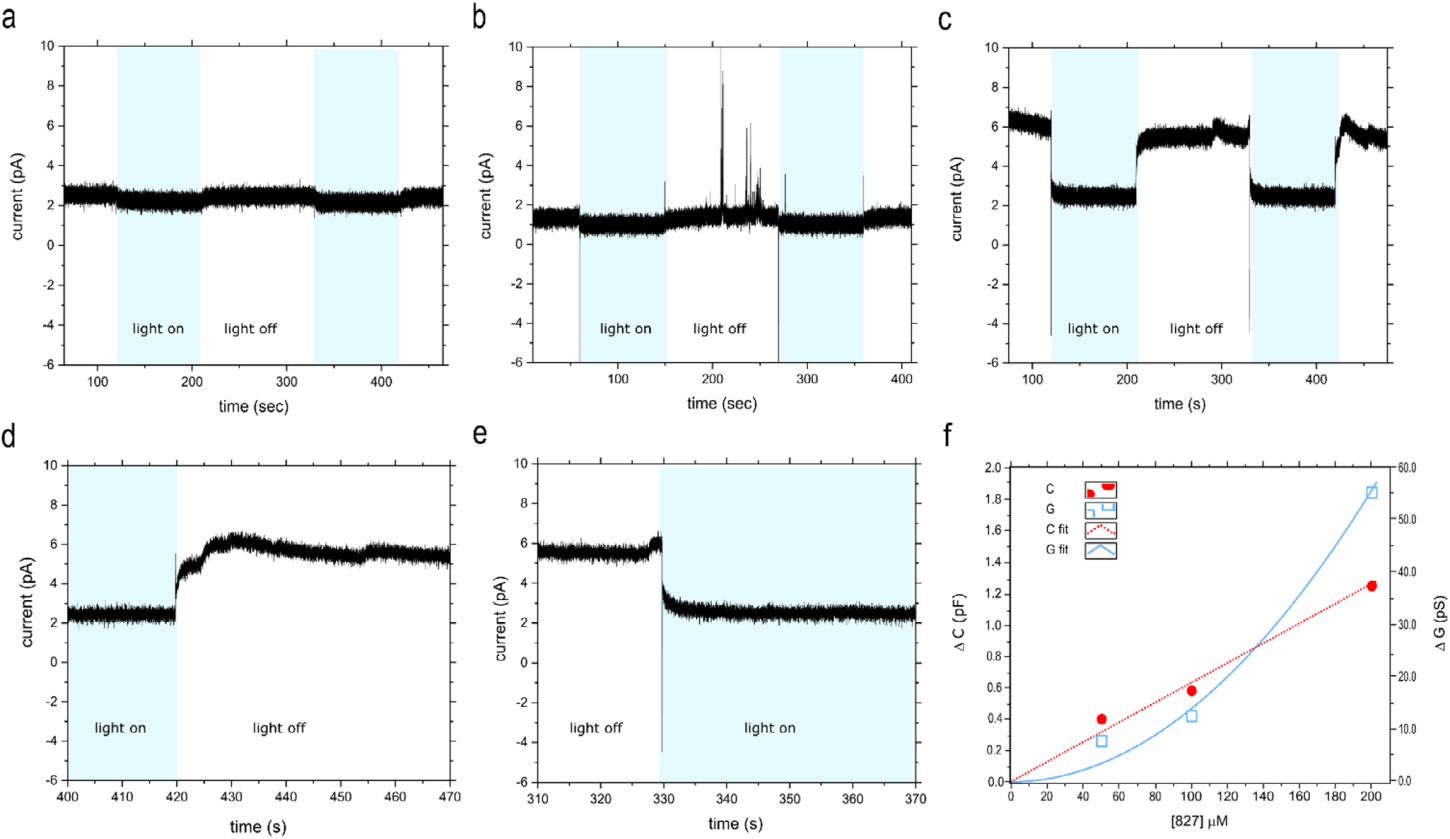
(a–c) Time trace of the current in DC experiments with 50 (a), 100 (b), and 200 µm (c) of **TC827** molecule, under alternating laser on and off. Time spans of 488 nm laser exposure at 4 mW are indicated by shaded light‐blue areas. (d) Zoom‐in of the switching‐off transient observed in panel (c). (e) Zoom‐in of the switching‐on transient from panel (c). (f) Laser‐induced steps in both the capacitance (ΔC, red filled circles) and conductance (ΔG, blue empty squares) extracted from AC (alternate current) experiments. The steps are evaluated for three different concentrations: 50, 100, and 200 µm. The red dashed line is a linear fit to the capacitance while the blue solid line is guide for the eyes, demonstrating the different non‐linear behavior.

The DC experiments (Figure 4) revealed an increase in the current when the light was switched off, whereas when the light was switched back on, the current decreased to a value close to the baseline. Several laser on/off cycles were performed to assess the reproducibility of the effect, which was confirmed a number of times.

The increase in current in dark conditions in response to different doses of the molecule was evaluated, revealing a larger effect at higher concentration of the photoactive molecule. Practically, the concentration of 50 µm does not seems to have significant effects on our system, since the variations induced at that dose are essentially within the noise level (Figure 4a). At the intermediate concentration of 100 µm (Figure 4b), the current exhibited a positive step of approximatively 0.3 pA in the dark; this response does not differ significantly from that observed at 50 µm, suggesting that the intermediate concentration is still insufficient to produce a measurable effect. At 200 µm (Figure 4c), a three‐fold increase in the current in the dark (up to ∼6 pA) was observed, followed by a complete return to its initial value (∼2 pA), suggesting a strong nonlinear dependence on concentration and the presence of a threshold‐like behavior.

In contrast to pore‐mediated photolipid responses, the current traces did not display stochastic current jumps or irreversible leakage currents, indicating that the conductivity increase is not caused by membrane disruption or pore formation.

While the change in current was the same for both light switch‐on and switch‐off, the dynamics of the process differed significantly. When the laser was switched off, the current increased in approximately 10 s (Figure 4d), whereas when the laser was turned back on, the current decreased within only 1–2 s (Figure 4e), accompanied by a very fast transient signal (on the millisecond timescale), which could be related to rapid charges displacement occurring during molecular photoisomerization.

These observations clearly indicate that the photoisomerization of the molecule into the lipid membrane affects the current in our device, although this peculiar behavior, to the best of our knowledge, has never been reported before in photolipids [17, 31, 32, 33].

This modulation of the current can be attributed to a cross‐membrane charge transport phenomenon favored by the molecule in the trans configuration. Indeed, it is likely that the molecules on the two sides of the bilayer, interacting with each other, allow charge tunnelling by virtue of the π‐conjugated structure of **TC827** molecule [34], thereby forming a sort of “light‐controlled molecular wire” [16].

**FIGURE 5:**
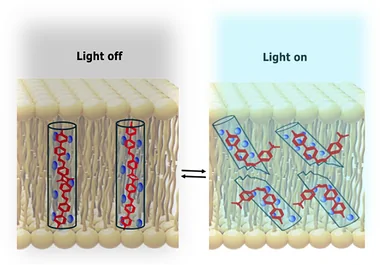
Schematic representation of the membrane lipid bilayer illustrating the proposed working mechanism. In dark condition, the molecules adopt the trans isomer configuration and align to form a transmembrane molecular wire, which is disrupted upon light irradiation as a result of molecular isomerization.

This occurs mainly at high concentration (200 µm), when enough molecules are present to form these conductive wires. The “wires” are disrupted when the molecule is in its cis form under illumination and, consequently, transmembrane transport is inhibited [34]. A schematic representation of the proposed working mechanism is shown in Figure 5.

The hypothesized mechanism also clarifies the different temporal dynamics observed (Figure 4d,e). When the light is turned off, the establishment of the “molecular wire” likely requires both the relaxation of **TC827** molecules to their trans isomeric form and the interaction between molecules embedded in both leaflets of the membrane—possibly involving their migration across the membrane and cooperative reorganization into conductive assemblies, thereby restoring the charge transport functionality. Therefore, a longer timescale is required, which could explain the gradual increase in current observed over several seconds. Conversely, upon laser irradiation, **TC827** rapidly switch to their cis configuration, promptly disrupting the conductive pathways across the membrane and causing the current to decrease within 1–2 s. Notably, when molecules were added only to the upper microchamber at a concentration of 200 µm, a very small current increase was observed in the dark (Figure S10), suggesting that the molecules are able to flip from the upper leaflet of the membrane to the opposite leaflet, although only to a limited extent.

We observed that both the conductance (G) and capacitance (C), extracted from the AC experiments (Figure 4f), increase in the dark. The changes induced by switching off the laser are shown in Figure 4f for different concentrations. Interestingly, the conductance increase (Figure 4f, blue curve) does not follow a linear trend with TC827 concentration. Consistent with the DC measurement described before (Figure 4a–c and Figure S11), this behavior suggests either a quadratic (or higher‐order) trend or the presence of a threshold effect. Both scenarios indicate that the observed current changes are unlikely to arise from individual molecules acting independently, but rather due to a cooperative mechanism involving the interaction of two or more **TC827** molecules [35, 36], supporting the hypothesis described above. In contrast, the increase in capacitance (Figure 4f, red curve) follows a linear trend with the concentration, suggesting that membrane capacitance depends on the intrinsic chemical structure of the single **TC827** molecules, which could alter the charge distribution at the membrane surface in the cis and trans configuration, affecting the membrane dielectric constant [30].

We would like to highlight that the behavior of molecule **TC827** is totally different from what has been previously reported for photo‐lipids or membranes embedding complex azobenzene molecules [17, 22]. Those systems typically show an increase in current only under illumination, when the molecules adopt the cis configuration. In such cases, the current increase is generally associated with membrane packing disruption, transient pore formation, and increased permeability [22, 25, 26, 31, 37].

Unlike the lipid‐like molecules with long organic chains reported [25, 38], our new molecule is rather short and operates through a different working principle, never observed before: it produces an enhancement of the current in the dark, when the trans configuration allows molecules from opposing leaflets of the membrane to establish conductive, light‐controlled molecular wires through their planar π‐conjugated chemical structure. On the other hand, in cis configuration the typical perturbation caused by lipid‐like molecules previously reported is not present; hence, the current remains low because no molecular pores are formed.

This distinction is central to the working mechanism of **TC827**: the molecule does not act by opening conductive defects in the membrane, but rather by promoting charge transport through light‐controlled molecular wires across an intact bilayer. Therefore, the increase in conductivity is not associated with membrane destabilization, as the membrane preserves its structural integrity. As a result, the device exhibits improved operational reliability and greater stability compared to previously reported systems.

### 2.3 Molecular Docking Simulation

To corroborate our hypothesis and to investigate the interaction of molecule **TC827** with the artificial membrane, we performed molecular docking simulations [39], to test the binding capacity of this molecule in either the trans or cis conformation. In the calculations, the assembly of DPHPC molecules was extracted from a hydrated bilayer previously equilibrated in a full‐atom molecular dynamics (MD) simulation with periodic boundary conditions [40]. Molecular docking is a “static” technique less powerful than MD simulation, and less commonly applied to biological membranes compared to protein systems, although it has already been used and experimentally validated for identifying binding modes of ligands to lipid bilayers [41, 42, 43]. In our case, reproducing the self‐assembling process of trans isomers of **TC827** into a “molecular wire” is too complex, whereas performing an MD simulation of a pre‐formed one would require making some strong initial assumptions on its geometry in terms of positions and orientations of individual molecules. In contrast, molecular docking has the advantage of providing in this case a blind prediction that does not rely on any arbitrary assumption that might bias the results.

**FIGURE 6:**
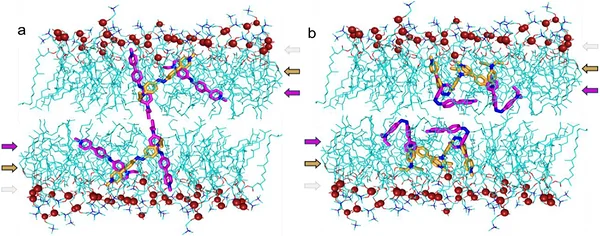
Simulated docking of molecule **TC827** conformations to the DPHPC membrane. Representative binding modes of molecule **TC827**, (a), in trans, and (b) in cis configurations. The color of the docking poses and the arrows indicate the approximate depth of association within the membrane: (gray) lipid head groups, (yellow) polar/apolar interface, and (magenta) hydrocarbon chains.

As shown in Figure 6, the azobenzene derivative **TC827** was never observed to be in contact with water in the docking simulations. In fact, the docking poses in trans conformation were found deeply plunged in the hydrocarbon region of the membrane (Figure 6a) or, at most, closer to the polar/apolar lipid interface. The binding energy to the membrane was also markedly favorable (−7.6 kcal/mol), indicating a higher affinity of **TC827** molecule for the lipid bilayer. These observations agree with the experiment, as the water/octanol partition of the molecule **TC827** indicated an almost exclusive preference (∼95%) for the apolar solvent (LogP = 1.3). A slight preference was observed for the pyridine ring of the compound to be closer to the polar region of the membrane; in these cases, the charged nitrogen atom could interact with the phosphate group of a lipid (as shown in Figure 6a in yellow). Moreover, the simulations suggest that the conformation of molecule has a sufficient length to promote the interaction between the aromatic rings in the apolar region, allowing the molecule to position itself within the membrane in agreement with the “molecular wire” hypothesis, although most of the docking positions of compound **TC827** did not show any distinct preference in the insertion direction, being exclusively in contact with the hydrocarbon chains of the lipids.

Simulations were also performed with the molecule **TC827** in cis conformation. As shown in Figure 6b, the results in this case showed that the molecules tend to plunge deeper into the lipid membrane in the regions of the hydrocarbon chains. The binding affinity was less favorable (by 0.3 kcal/mol) compared to the molecule in the trans form, although this energy value is close to the uncertainty due to the accuracy of the scoring function of the docking algorithm (0.2‒0.3 kcal/mol).

All these findings are in accordance with the electrical measurements. In the case of the cis conformation, conformational changes of molecule **TC827** under light irradiation could cause the interruption of the “molecular wire,” with the restoration of the bilayer's initial properties. It could be noted that the observed differences in the binding location and affinity of molecule **TC827** in the two distinct conformers, cis and trans, may also alter in subtle ways some other of properties of the lipid assembly (e.g., fluidity, polarity, etc.), ultimately further affecting the macroscopic properties of the membrane.

## 3 Conclusions

The present study introduces a novel photoswitchable molecule, **TC827,** that modulates the electrical behavior of an artificial lipid membrane through a new working mechanism, distinct from pore‐mediated permeability changes. Differently from conventional azobenzene derivatives, **TC827** facilitates charge transport in its trans state by forming “light‐controlled molecular wires” that increase conductivity in the dark. This mechanism is disrupted upon light irradiation, reverting the molecule to its cis configuration.

These findings underscore the potential of short, non‐lipid‐like photochromic molecules as versatile components for modulating membrane properties in optoelectronic devices, providing new avenues for biointerface engineering and photo‐responsive systems and opening opportunities for the control of living systems or pharmacological delivery.

By bridging the gap between organic bio‐materials and optoelectronic technology, our research may contribute to developing sustainable, high‐performance devices that open new opportunities for developing next‐generation photonic sensors, such as optical switches or biomimetic optical systems based on biological materials.

## 4 Methods

### 4.1 Chemicals: Lipids, Solvents, Electrolyte Solutions, and Molecules

1‐palmitoyl‐2‐ [3‐(4‐((1E,3E,5E)‐6‐phenylhexa‐1,3,5‐trien‐1‐yl)phenyl)propanoyl]‐sn‐glycero‐3‐phosphocholine (DPhPC 16:0) and DMPC (1,2‐dimyristoyl‐sn‐glycero‐3‐phosphocholine) were purchased from Avant Polar Lipids, part of CRODA international Plc. n‐Octane, Methanol, Ethanol, Toluene solvents were purchased from Sigma–Aldrich at 99% purity, and used as received. The KCl solution was prepared at 1 M stock dissolving the powder purchased from Sigma–Aldrich in MilliQ water. A silver wire was used for the Ag/AgCl electrodes preparation. The Chlorination was performed using commercial bleach. The plasmid used for the MSP1E3D1 was provided by Addgene 20066.

The ^1^H and ^13^C NMR spectra were recorded on a Bruker AVANCE III 400 MHz instrument using chloroform‐*d* (CDCl_3_) and methanol‐*d_4_* (CD_3_OD) as solvents, splitting patterns were described as singlet (s), doublet (d), triplet (t), quartet (q), or multiplet (m). Elemental analyses were done by a Carlo Erba CHNS–O EA1108‐Elemental Analyzer. Mass spectroscopy was recorded using a LC‐MS‐Agilent.

### 4.2 Photoswitching Molecule Synthesis

All starting materials were purchased from commercial sources and used without further purification. All solvents and reagents were used as received, unless otherwise claimed. The 1H and 13C NMR spectra were recorded on a Bruker AVANCE III 400 MHz instrument using chloroform‐d (CDCl3) and methanol‐d4 (MeOD‐d4) as solvents, and splitting patterns were described as singlet (s), doublet (d), triplet (t), quartet (q) or multiplet (m). Elemental analyses were done by Carlo Erba CHNS–O EA1108‐ Elemental Analyzer. LC‐MS spectra were acquired with an Agilent 6300 Series Ion Trap source (APCI).

#### 4.2.1 Synthesis of (E)‐4‐((4‐bromophenyl)diazenyl)‐N,N‐diethylaniline, **1**

4‐Br‐aniline (0.012 mol, 2.00 g) was dissolved in tetrafluoroborate acid (48% water, 10 mL) at 0°C, resulting in a milky solution. Sodium nitrite (0.014 mol, 1.00 g) was carefully added, the mixture was stirred at 0°C for 40 min. Diethylaniline (0.024 mol, 3.58 g) was added, and the mixture was stirred at room temperature for 2 h. The pH of the mixture was neutralized by adding NaOH, obtaining the product precipitation. The residue was filtered, dissolved in minimum DCM, and then purified by column chromatography (silica gel 60, 0.2 mesh) using hexane/EtOAc (2:1, respectively) as an eluent to afford the product in 92% yield. ^1^H NMR (400 MHz, Chloroform‐*d*) δ 7.86 (d, *J* = 8.8 Hz, 2H), 7.71 (d, *J* = 8.6 Hz, 2H), 7.57 (s, 2H), 6.73 (s, 2H), 3.46 (q, *J* = 7.1 Hz, 4H), 1.24 (t, *J* = 7.1 Hz, 6H). ^13^C NMR (101 MHz, CDCl_3_) δ 152.06, 150.43, 143.04, 132.11, 125.57, 123.71, 123.09, 111.08, 44.82, 12.66. LCMS (APCI) Calcd for C_16_H_18_BrN_3_ [M + H] ^+^ 333,24; found 333,26. Anal. Calcd for C_16_H_18_BrN_3_:  C, 57.84; H, 5.46; Br, 24.05; N, 12.65. Found:C, 56.40; H, 5.52; Br, 24.17; N, 13.91.

#### 4.2.2 Synthesis of (E)‐N,N‐diethyl‐4‐((4‐(pyridin‐4‐yl)phenyl)diazenyl)aniline, **2**

In the microwave vessel reactor tube was placed a mixture solvents of toluene (10 mL) H_2_0 (5 mL) and ethanol (5 mL). To it was added compound **1** (1.20 mmol, 400 mg), pyridin‐4‐ylboronic acid (1.45 mmol, 177 mg), and Na_2_CO_3_ (16 mmol, 1.7 g). The mixture was bubbled with argon for 1.5 h then Pd(Ph_3_)_4_ (24.1 µmol, 25.4 mg) was added. The reaction was assisted microwave (temperature 80°C), after 50 min the mixture was cooled to r.t., diluted with ethyl acetate and washed with H_2_O. The organic layer was dried over Na_2_SO_4_, filtered, and concentrated in vacuo. The product was purified by silica gel column chromatography using hexane‐EtOAc (10:1) as eluent. Compound 2 was obtained as an orange powder in 84% yield. ^1^H NMR (400 MHz, Chloroform‐*d*) δ 8.68 (d, *J* = 5.9 Hz, 2H), 7.94 (d, *J* = 8.6 Hz, 1H), 7.89 (d, *J* = 9.2 Hz, 2H), 7.76 (d, *J* = 8.6 Hz, 2H), 7.58 (d, *J* = 6.1 Hz, 2H), 6.74 (d, *J* = 9.2 Hz, 2H), 3.47 (q, *J* = 7.1 Hz, 4H), 1.25 (t, *J* = 7.1 Hz, 6H). ^13^C NMR (101 MHz, CDCl_3_) δ 153.52, 150.23, 149.95, 147.63, 143.01, 138.06, 127.43, 125.42, 122.66, 121.31, 110.81, 44.56, 12.48. LCMS (APCI) calcd for C_16_H_18_BrN_3_ [M + H] ^+^ 331,43; found 333,45. Anal. Calcd for C_21_H_22_N_4_: C, 76.33; H, 6.71; N, 16.96. Found:C, 78.56; H, 6.65; N, 14.83.

#### 4.2.3 Synthesis of (E)‐4‐(4‐((4‐(diethylamino)phenyl)diazenyl)phenyl)‐1‐methylpyridin‐1‐ium iodide, **TC827**

Compound **2** (500 mg, 1.66 mmol) was dissolved in anhydrous acetonitrile (5 mL). Methyl iodide (33.2 mmol, 4.71 g) was added dropwise to the reaction mixture which was stirred at r.t. for 12 h. The precipitate was filtered, washed with acetonitrile and diethyl ether to remove excess methyl iodide and then dried in vacuo. Compound **3** was obtained as an orange solid in 98% yield. ^1^H NMR (400 MHz, Methanol‐*d*4) δ 8.87 (d, *J* = 6.8 Hz, 2H), 8.44 (d, *J* = 7.0 Hz, 2H), 8.15 (d, *J* = 8.8 Hz, 2H), 8.00 (d, *J* = 8.8 Hz, 2H), 7.87 (d, *J* = 9.3 Hz, 2H), 6.84 (d, *J* = 9.3 Hz, 2H), 4.90 (s, 3H), 4.40 (s, 1H), 3.54 (q, *J* = 7.1 Hz, 2H), 3.35–3.27 (m, 4H), 1.25 (t, *J* = 7.1 Hz, 6H). ^13^C NMR (101 MHz, MeOD) δ 155.35, 155.17, 151.02, 144.98, 142.90, 133.27, 128.57, 125.56, 123.88, 122.57, 110.68, 46.34, 44.14, 11.32. LCMS (ESI) calcd for C_22_H_25_N_4_^+^ [M] ^+^ 345,46; found 345,48.

### 4.3 Device Features and Planar Lipid Bilayers

The acellular device used in this work consisted of a commercial microfluidic device (Ionovation Bilayer Explorer V02, Ionovation GmbH) comprising two compartments of approximately 150 µL each, separated by a PTFE septum with a 100–120 µm microhole. The upper chamber has three openings on the top surface and no internal constrictions. The lower chamber is characterized by an additional triangular channel connected to a larger compartment comparable in size to the upper chamber. It is also connected to two openings on the top surface, allowing it to be filled with the electrolyte solution. The lower chamber is isolated from the upper chamber except through the microhole in the septum. The artificial membrane was produced by the air bubble technique into a commercial microfluidic device. A drop of 10mg/ml of DPhPC solution in n‐octane (Sigma–Aldrich) was micropipetted in proximity of the micro‐hole. After its formation, the BLM was sitting horizontally between two micro chambers filled by the electrolyte solution (0.5 M KCl), each independently accessible and provided by electrodes. The lipid phase between the two water compartments underwent a spontaneous thinning process that ultimately yielded a single‐bilayer membrane. This formation was monitored optically and by capacitance measurements. The latter were performed by measuring the electric current between the chambers upon applying a triangle wave voltage (peak‐to‐peak amplitude 100 mV, frequency 10 Hz). The Ag electrodes were chlorinated by immerging them in a bleach solution. After the Ag/AgCl electrodes were directly immersed in the electrolite solution througth two holes connected to each chamber, they were also connected to the head stage of a current amplifier (Low Noise Current Preamplifier – Standford Research System). The amplified currents were digitized at a sampling interval of 1 ms and fed into a converter (National Instrument, 8 inputs, 16‐Bit, 2.0 MS/s X Series Multifunction DAQ) for acquisition and storage of the signal on a personal computer. For analysis, a Windows‐based analysis software (LabView) developed in our laboratory was used in combination with OriginPro 2018 software (Microcal Software Inc.).

We estimated the membrane thickness from the measured capacitance values using the capacitor model (C = ε_0_εA/d) [44]. We considered the measurements performed on a typical BLM in the absence of molecules (Figure 2c) with the diameter of ∼120 µm (excluding the annulus), which corresponds to an area of approximately 11310 µm^2^. From the measured capacitance of approximately 50 pF and assuming a dielectric constant ε = 2.2 [45], we obtain:

$$d=\frac{\varepsilon_{0}\varepsilon A}{C}=\;\frac{8.85\cdot 10^{-12}\;\cdot 2.2\;\cdot 1.131\cdot 10^{-8}}{50\cdot 10^{-12}}=4\cdot 10^{-9}m$$

The estimated membrane thickness is therefore approximately 4 nm, which is in good agreement with typical lipid bilayer thickness values reported in the literature.

In the AC experiments, we evaluate both the capacitance C and conductance G by assuming that the total current is due to a capacitive current and a resistive current. The expression with the two terms reads I = C*(dV/dt) + G*V, where I is the measured current and dV/dt is the voltage slope in the triangular waveform. C and G are extracted by fitting the linear region of the IV plot.

### 4.4 Nanodiscs and Liposome Formation

For Nanodiscs formation, a DMPC stock was prepared by suspending lipids in milli‐Q water up to a final concentration of 100 mM. For each individual lipid, a particular final concentration of sodium cholate was required in order to obtain a clear suspension; in this case, 200 mm of sodium cholate were used. The MSP1E3D1 scaffold proteins were expressed in E.coli BL21 (DE3) transformed with Addgene plasmid 20066 as previously described [27]. This protein had N‐terminal poly‐histidine tags used for its purification using affinity chromatography with HisPur Ni‐NTA Resin (Thermo scientific), after that it was stored at 4°C. The MSP1E3D1 construct and full amino acid sequences are available as Supporting Information. Concentrations were determined from the absorbance at 280 nm using calculated extinction coeficients [46]. All buffers consisted of 10 mm Tris–HCl, pH 7.4, 0.1 M NaCl, 0.01% NaN3. Water was purified with a Milli‐Q system. For ND assembly [27, 47, 48, 49, 50] the MSP1E3D1 was mixed with DMPC and cholate at a molar ratio of 1:80 and incubated for >2 h at 24°C, followed by detergent removal with a XAD‐2 resin. Amberlite XAD‐2 was obtained from Serva, Heidelberg. The Amberlite beads were washed three times with methanol and then equilibrated and stored in distilled water. The amount of XAD‐2 necessary for the detergent removal was estimated according to the detergent concentration, and was calculated experimentally as described previuosly [50, 51]. For sodium cholate removal, the ratio used was 1 mg of XAD per 8 mmol of sodium cholate, approximatively.

### 4.5 N‐octanol/Water Partition Coefficient

The partition coefficient was calculated by adding the molecule 827 in a water solution and then it was put in contact with the octanol solvent. The two liquid compartments were mixed and after that it was evaluated the incorporation of the molecule in the octanol phase. The final results were evaluated by UV–vis spectroscopy.

### 4.6 Photoswitching Electrical Measurements

**FIGURE 7:**
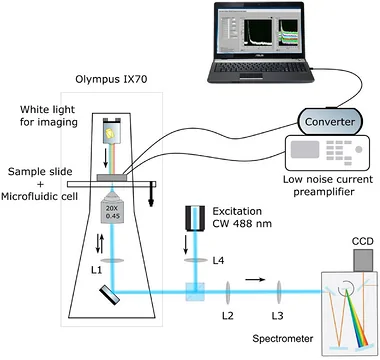
Optical and electrical setup used for the measurement; the light blue line is the laser at 488nm that passes through different lenses and mirrors that allow the entrance of the laser into the microscope objective and its arrival directly on the flowcell hole and so on the membrane. The laser is then reflected into the camera, which makes it possible to simultaneously visualize the membrane and the laser to determine the correct position.

Membrane currents were measured with a pre‐amplifier (Low Noise Current Preamplifier – Standford Research System) and a current generator (peak‐to‐peak amplitude 100 mV, frequency 10 Hz). The current‐voltage data were taken after the membrane capacitance was in the range from 20 to 50 pF, in accordance with the characteristics of the membrane (diameter and thickness) and the conductivity reached a constant level. This was evaluated by monitoring the current when a 100 mV voltage was applied either continuously or alternately across the membrane. Generally, Silver wire was used as electrodes, which were chlorinated to obtain Ag/AgCl electrodes. All measurements were carried out at room temperature. The molecule was dissolved in methanol at a concentration of 2 mm; 10 µL were added to the upper and lower chamber in order to obtain a final concentration of 200 µm. The optical part consists of an optical microscope (Olympus 1×2‐UCB) through which it can visualize and excite the membrane. Using an optical path on an optical table, the microscope image was projected onto the entrance slits of an imaging spectrometer (Horiba, iHR320) equipped with a 300 lines/mm grating and coupled to a 2D enhanced charge‐coupled device (Hamamatsu, EMCCD C9100). It is possible to obtain imaging of the membrane (using a halogen broadband white light source in a transmission configuration) and, simultaneously, align the laser (in reflection configuration) to direct the beam to the center of the membrane. A lateral aperture in the microscope allows the laser to be coupled and aligned through a series of mirrors and lenses (ThorLabs Inc.) (Figure 7).

The laser beam is then focused onto the membrane using a 20X objective (Olympus) in a spot size with a Full Width at Half Maximum (FWHM) comparable to the hole in which the membrane is formed. We used a continuous‐wave (cw) Diode Laser at 488nm (MKS‐SpectraPhysics). For time‐resolved measurements, the opening and closing time of the laser was controlled by using an optical shutter (Thorlabs, SH05R) interfaced with a computer and integrated into a custom‐designed LabView program.

### 4.7 Photoswitching Spectroscopic Measurement on PMMA Films

The molecule synthesis was performed in the Chemistry laboratories at CNR‐Nanotec. The PMMA (Mw:120 000) was purchased from Sigma–Aldrich and used without further purification. The molecular structure of the dyes is shown in Figure 1. The dyes and polymer were dissolved in 9:1 solution of Toluene and Methanol with a concentration of 20 µm for each component. Films were formed from 100 µL drop of solution with a spin coating of 3000 rpm on a glass substrate (1 cm × 1 cm), giving clear films having 20 µm samples were dried on a hot plate at 100°C for 20 min.

**FIGURE 8:**
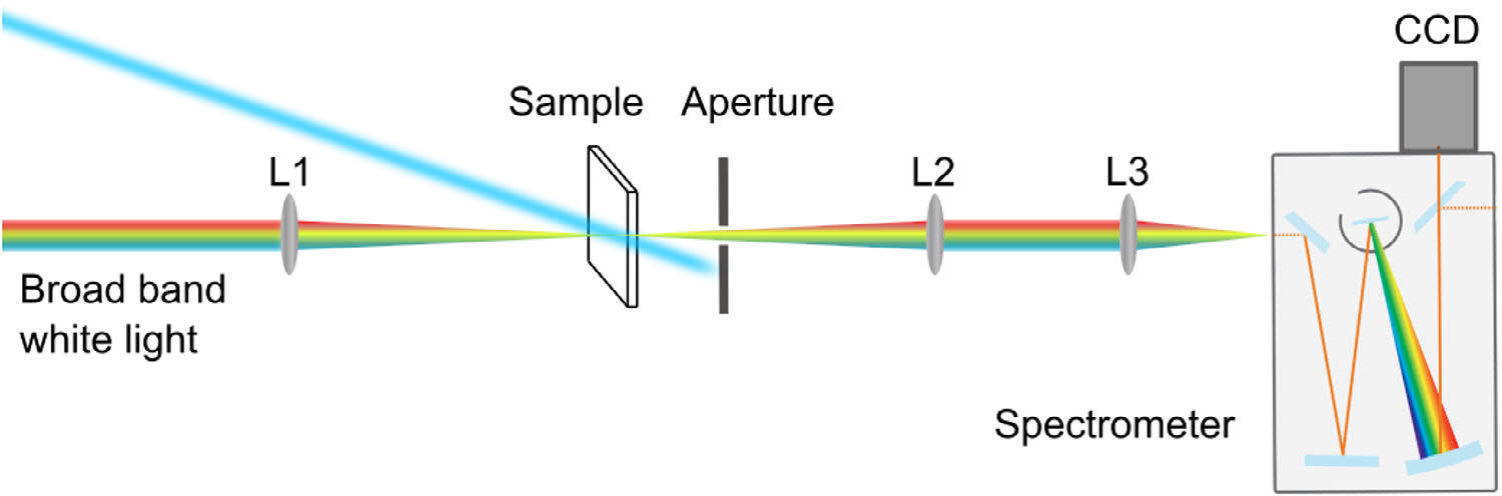
Schematic representation of the optical setup used for the photoswitching absorption spectrum before, after, and during irradiation with a 488nm diode laser.

The instrument for in situ absorption measurement is shown in Figure 8, where a broadband halogen lamp was used as a source and a spectrometer (Princeton Instruments, Action Spectra Pro SP‐2300) was employed. The spectrometer is equipped with three gratings (150, 300, and 600 lines/mm) and coupled to a 2D Charge‐coupled device (Princeton Instruments, Pixis 400). Minimum temporal resolution for the system was about 20 ms, but typically used resolution are in the order of about 1 s due to integrating spectra for better quality. Under our experimental conditions, spectral measurements were taken every 5 s. Absorbance spectra along time were obtained by dividing the spectra of the transmitted light passing through the PMMA + molecule film by a reference spectrum collected on the bare film (glass + PMMA without molecule).

For excitation, a linearly polarized 488 nm cw laser was used. Its intensity was distributed in a nearly Gaussian shape with a FWHM of 3 mm. The laser excites the molecule nearly at the maximum absorption and hits the sample at an angle of roughly 30 degrees. The broadband light is used to track the spectral modifications in the absorption induced by the pumping laser and is focused in a spot with an FWHM of 2 mm. The two beams are overlapped in space on the sample plane and the laser is removed along the detection line filtering with an iris aperture. An electromechanical shutter was used to control the laser irradiation on the sample, and measurements were made during time of laser exposure of 1 min and about 10 min after closing the shutter [52].

For the spectroscopic measurements in bulk solutions, the molecules were dissolved in methanol and added to 50% ethanol and a 0.2 M NaCl solution to reach a concentration of 25 µm. The measurements were taken using the NanodropOne spectrophotometer (Thermo Scientific) and the irradiation source was a white light lamp.

### 4.8 Molecular Docking Simulation

A model of DPHPC membrane containing two layers of 6 × 6 lipids and 2953 water molecules, equilibrated in MD simulation for 50 ns at the temperature of 303.15 K in the isothermal‐isobaric ensemble by using the force field CHARMM [40], was downloaded in the Protein Data Bank format. Molecular structures of compound was built by using the chemical editor software Avogadro, version 1.2.0 [53]. All preliminary operations, including merging apolar hydrogens to the carbon atoms they were attached to, were carried out by using the software suite AutoDock Tools, version 1.5.6 [54]. Water molecules were eliminated from the DPHPC membrane, according to standard practices. The compound was modelled either in trans or cis conformation by constraining the rotation around the N = N dihedral angle. Full flexibility was otherwise guaranteed for all the other bond torsions, resulting in six rotational degrees of freedom for molecule 827. Molecular docking simulations were performed by using the software AutoDock Vina, version 1.2.5 [39], following a protocol previously employed [55]. To increase the variety of potential binding locations, a single different lipid molecule in the central region of the DPHPC membrane was deleted in each additional simulation run, mimicking possible displacements of lipids in the membrane due to the binding of the compounds. The docking search was performed on the region centred on a single lipid monolayer, for both monolayers separately, and within a volume of 40 × 40 × 30 Å in each case.

## Author Contributions

I.C., D.S., and L.D.M. conceived the idea and planned the work. D.S. and L.D.M. coordinated the experimental work. I.C. performed the partition experiments, the spectroscopic analysis in solution, the optimization of artificial membrane formation, the photoelectrical measurements on microfluidic device. A.F., L.D., and I.C. conducted the spectroscopic characterization on PMMA‐films. A.C. built the electrical setup and, with the help of L.D., supervised photoelectric measurements. V.A., A.F., D.B., L.D., and M.D.G implemented the optical setup. V.A. and D.S supervised optical measurements. L.D. developed the software program for automating and analyzing photoelectrical measurements. A.L.C designed and carried out the synthesis and characterization of **TC827**. B.R. performed molecular simulations. I.C. and L.D. performed the analysis on the direct current (DC) and alternate current (AC) data. I.C. and L.D.M wrote the first draft of the manuscript. All authors contributed to the discussion, interpretation of the data, and revised the manuscript.

## Conflicts of Interest

The authors declare no conflicts of interest.

## Supporting information




**Supporting File**: advs77303‐sup‐0001‐SuppMat.docx.

## Data Availability

The data that support the findings of this study are available in the supplementary material of this article.
