# Supplementary material for: Light‐Controlled Conductive Molecular Wire in an Artificial Lipid Membrane
Source: Adv Sci (Weinh). 2026 Aug 21;13(52):e77303. doi: 10.1002/advs.77303 (PMC13496156; doi:10.1002/advs.77303)
Supplement: Supplementary file 1 [file ADVS-13-e77303-s001.docx]

**Light-controlled conductive molecular wire in an artificial lipid membrane**

***Ilaria Cardace^1^, Lorenzo Dominici^1^, Adriano Cola^2^, Antonio Fieramosca^1^, Vincenzo Ardizzone^1^, Agostina Lina Capodilupo^1^, Bruno Rizzuti^3,4^, Milena De Giorgi^1^, Dario Ballarini^1^, Giuseppe Gigli^5^, Daniele Sanvitto^1^*, Luisa De Marco^1^****

*^1^CNR-NANOTEC, Institute of Nanotechnology, Lecce (LE), Italy; ^2^CNR-IMM, Institute for Microelectronics and Microsystems, Lecce (LE), Italy; ^3^CNR-NANOTEC, Institute of Nanotechnology, Rende (CS) secondary site, Italy; ^4^Institute of Biocomputation and Physics of Complex Systems, University
of Zaragoza, Zaragoza, Spain; ^5^Department of Experimental Medicine, University of Salento, c/o Campus Ecotekne, via Monteroni, Lecce, Italy.*

**e-mail:* [luisa.demarco@cnr.it](mailto:luisa.demarco@cnr.it); [daniele.sanvitto@cnr.it](mailto:daniele.sanvitto@cnr.it)

**SUPPLEMENTARY INFORMATION**

**Figure S1. Synthetic route of compound TC827.**

Synthesis of (E)-4-((4-bromophenyl)diazenyl)-N,N-diethylaniline, **1**.

4-Br-aniline (0.012 mol, 2.00 g) was dissolved in tetrafluoroborate acid (48% water, 10 mL) at 0 °C, resulting in a milky solution. Sodium nitrite (0.014 mol, 1.00 g) was carefully added, the mixture was stirred at 0 °C for 40 min. Diethylaniline (0.024 mol, 3.58 g) was added, and the mixture was stirred at room temperature for 2 h. The pH of the mixture was neutralized by adding NaOH, obtaining the product precipitation. The residue was filtered, dissolved in minimum DCM, and then purified by column chromatography (silica gel 60, 0.2 mesh) using hexane/EtOAc (2:1, respectively) as an eluent to afford the product in 92% yield. ^1^H NMR (400 MHz, Chloroform-*d*) δ 7.86 (d, *J* = 8.8 Hz, 2H), 7.71 (d, *J* = 8.6 Hz, 2H), 7.57 (s, 2H), 6.73 (s, 2H), 3.46 (q, *J* = 7.1 Hz, 4H), 1.24 (t, *J* = 7.1 Hz, 6H). ^13^C NMR (101 MHz, CDCl_3_) δ 152.06, 150.43, 143.04, 132.11, 125.57, 123.71, 123.09, 111.08, 44.82, 12.66. LCMS (APCI) calcd for C_16_H_18_BrN_3_ [M + H] ^+^ 333,24; found 333,26. Anal. Calcd for C_16_H_18_BrN_3_:  C, 57.84; H, 5.46; Br, 24.05; N, 12.65. Found*:*C, 56.40; H, 5.52; Br, 24.17; N, 13.91.

Synthesis of (E)-N,N-diethyl-4-((4-(pyridin-4-yl)phenyl)diazenyl)aniline, **2**.

In the microwave vessel reactor tube was placed a mixture solvents of toluene (10 mL) H_2_0 (5 mL) and ethanol (5 mL). To it was added compound **1** (1.20 mmol, 400 mg), pyridin-4-ylboronic acid (1.45 mmol, 177 mg) and Na_2_CO_3_ (16 mmol, 1.7 g). The mixture was bubbled with argon for 1.5 h then Pd(Ph_3_)_4_ (24.1 μmol, 25.4 mg) was added. The reaction was assisted microwave (temperature 80°C), after 50 minutes the mixture was cooled to r.t., diluted with ethyl acetate and washed with H_2_O. The organic layer was dried over Na_2_SO_4_, filtered, and concentrated in vacuo. The product was purified by silica gel column chromatography using hexane-EtOAc (10:1) as eluents. The compound 2 was obtained as an orange powder in 84% yield. ^1^H NMR (400 MHz, Chloroform-*d*) δ 8.68 (d, *J* = 5.9 Hz, 2H), 7.94 (d, *J* = 8.6 Hz, 1H), 7.89 (d, *J* = 9.2 Hz, 2H), 7.76 (d, *J* = 8.6 Hz, 2H), 7.58 (d, *J* = 6.1 Hz, 2H), 6.74 (d, *J* = 9.2 Hz, 2H), 3.47 (q, *J* = 7.1 Hz, 4H), 1.25 (t, *J* = 7.1 Hz, 6H). ^13^C NMR (101 MHz, CDCl_3_) δ 153.52, 150.23, 149.95, 147.63, 143.01, 138.06, 127.43, 125.42, 122.66, 121.31, 110.81, 44.56, 12.48. LCMS (APCI) calcd for C_16_H_18_BrN_3_ [M + H] ^+^ 331,43; found 333,45. Anal. Calcd for C_21_H_22_N_4_: C, 76.33; H, 6.71; N, 16.96. Found*:*C, 78.56; H, 6.65; N, 14.83.

Synthesis of (E)-4-(4-((4-(diethylamino)phenyl)diazenyl)phenyl)-1-methylpyridin-1-ium iodide, **TC827**.

Compound **2** (500 mg, 1.66 mmol) was dissolved in anhydrous acetonitrile (5 mL). Methyl iodide (33.2 mmol, 4.71 g) was added dropwise to the reaction mixture which was stirred at r.t. for 12 hours. The precipitate was filtered, washed with acetonitrile and diethyl ether to remove excess methyl iodide and then dried in vacuo. Compound **3** was obtained as an orange solid in 98% yield. ^1^H NMR (400 MHz, Methanol-*d*4) δ 8.87 (d, *J* = 6.8 Hz, 2H), 8.44 (d, *J* = 7.0 Hz, 2H), 8.15 (d, *J* = 8.8 Hz, 2H), 8.00 (d, *J* = 8.8 Hz, 2H), 7.87 (d, *J* = 9.3 Hz, 2H), 6.84 (d, *J* = 9.3 Hz, 2H), 4.90 (s, 3H), 4.40 (s, 1H), 3.54 (q, *J* = 7.1 Hz, 2H), 3.35 – 3.27 (m, 4H), 1.25 (t, *J* = 7.1 Hz, 6H). ^13^C NMR (101 MHz, MeOD) δ 155.35, 155.17, 151.02, 144.98, 142.90, 133.27, 128.57, 125.56, 123.88, 122.57, 110.68, 46.34, 44.14, 11.32. LCMS (ESI) calcd for C_22_H_25_N_4_^+^ [M] ^+^ 345,46; found 345,48.

**Figure S2. ^1^H NMR (400 Hz, CDCl_3_) of compound 1.**

**Figure S3. ^13^C NMR (100 Hz, CDCl_3_) of compound 1.**

**Figure S4. ^1^H NMR (400 Hz, CDCl_3_) of compound 2.**

**Figure S5. ^13^C NMR (100 Hz, CDCl_3_) of compound 2**.

**Figure S6. ^1^H NMR (400 Hz, MeOD) of compound TC827.**

**Figure S7. ^13^C NMR (100 Hz, MeOD) of compound TC827**

**TC827 molecule partition into lipidic environments.** The trans form of the molecule has a linear geometry that favours the intercalation parallel to the phospholipid leaflets. The liposomes and nanodiscs were also used to evaluate this phase partitioning of the molecule between the lipid and water environment. For the first case, the **TC827** molecule dissolved in methanol solvent was added to the Giant unilamellar vesicles solution, and after that, the sample was centrifuged. The molecule tends to be incorporated into the lipidic insoluble fraction (Fig. S8a). A change of the absorption spectrum of the molecule is observed in the two phases, with a blue shift after the incorporation (Fig. S8b). The same experiment was performed in a more sophisticated system. The molecules dissolved in methanol were added to a solution of preassembled nanodiscs. They consist of a pair of helical amphipathic scaffold proteins (MSP1E3D1) and a central core of lipids. ^1–3^ They are useful tools in the study of membrane proteins, which are embedded in cell membranes. Lipid nanodiscs provide a more native-like environment because they simulate the cell membrane physical environment. ^4^ Also in this case, a blue shift is observed for the 827 molecule that is incorporated into the nanodiscs and so the lipidic environment (Fig. S8c). To separate the molecules that remain in solution from the ones intercalated into the Nanodiscs, the ion metal affinity chromatography was performed using a Nichel-agarose Resin. Indeed, the MSP1E3D1, which forms the external structure of the nanodiscs, is expressed with a Histidine-Tag at the N-terminus of the amino acid sequence. So, when the solution containing the nanodiscs with the **TC827** molecule is added to the resin, the MSP interact with it. The sample was centrifuged, and at the end of the pull-down experiment, two phases were present, one with the nanodiscs attached to the resin and the other with the solution that should be clear. The experiment was also performed without nanodiscs as a negative control, and the results were evaluated with UV-Vis spectroscopy. The absorbance spectrum without or with Nanodiscs at T0 (fig. S8c, respectively red dashed and continuous lines) and the supernatant, after 827-NDs pull down, were measured at the spectrophotometer (fig.S8c, respectively green dashed and continuous lines). In detail, there is a blue shift of the 827 molecules' absorbance spectrum when they interact with nanodiscs, presenting two characteristics isosbestic points around 495nm and 350nm. The molecules soluble in water, and so not intercalated into NDs, remain in the supernatant and reacquire the same absorbance spectrum of the molecule in solution without nanodiscs (fig. S8c, red and green dashed lines). The reduction of the peak is almost 90%. However, since it was demonstrated that the resin also has an affinity toward the 827 molecules, just 42% of the molecules in solution interact or intercalate into the nanodiscs membrane, and this value depends on the concentration of the nanodiscs in solution and on the number of molecules that could be incorporated into each nanodisc.

**Figure S8.** Image of visible 827 partition in the lipidic phase of a lipidic vesicle-water emulsion after centrifugation at 16000 rpm (**a**). Phase partitioning evaluation by absorption spectrum in 827 solution (200 µM) dye with or without Giant Lipid (DMPC) Vesicles (**b**) or with Nanodiscs (produced with MSPE3D1 and DMPC) at pH 8 (**c**), the samples measured were incubated with nanodiscs-His tagged for 1h before Ni-NTA pull down. (**d**) Phase partitioning evaluation by the absorption spectrum in the solution of the dye TC827 (20 µM) between water (in red) and octanol solution (in orange).

**Figure S9.** (**a-c**) Photoelectric measurement of the signal from the planar lipid bilayer upon application of a 100 mV AC (alternating current) triangular wave voltage at 10 Hz modulation frequency, after the **TC827** molecule addition at different concentrations: 50 µM (a), 100 µM (b), 200 µM (c). The laser switch on is indicated by the shaded light blue areas. (**d-f**) Magnification of modulated current along five periods after molecule addition in the dark at three different TC827 concentration: (**d**) 50 µM, (**e**) 100 µM, and (**f**) 200 µM. (**g**) Magnification of modulated current along ten periods of the 200 µM TC827 including the switching on of the laser which shows the slight decrease of the slope and the amplitude of the square-wave. (**h**) The current-voltage response (I-V) was analyzed over 100-ms cycles of the applied triangular wave voltage. A linear fit was applied to the positive (in blue) and negative (in red) semi-quarter of the waveform. The m slopes represent the resistance component (inverse of the conductance) while the intercept (q) corresponds to the capacitive response. (**i**) Capacitance time traces taken from the fit of the current waveform (Figure h) for 200 µM TC827 concentration. The exposure to light (light blue area) induces a slight decrease in membrane capacitance.

**Figure S10.** Time trace of the current in DC experiments with 200 µM of **TC827** molecule added only in the upper chamber of the microfluidic device.

**Figure S11.** Laser-induced steps in both the capacitance (ΔC, red filled circles) extracted from AC experiments and direct current (ΔI, blue squares). The steps are evaluated for three different concentrations: 50, 100 and 200 µM. The red solid line is a linear fit to the capacitance, while the blue solid line is a guide for the eyes.

**References**

1.	Mitra, N. Nanodiscs: Membrane Protein Research in Near-Native Conditions. *Materials and Methods* **3**, (2013).

2.	Skar-Gislinge, N., Johansen, N. T., Høiberg-Nielsen, R. & Arleth, L. Comprehensive Study of the Self-Assembly of Phospholipid Nanodiscs: What Determines Their Shape and Stoichiometry? *Langmuir* **34**, 12569–12582 (2018).

3.	Li, M. J., Atkins, W. M. & McClary, W. D. Preparation of Lipid Nanodiscs with Lipid Mixtures. *Curr Protoc Protein Sci* **98**, (2019).

4.	Hagn, F., Nasr, M. L. & Wagner, G. Assembly of phospholipid nanodiscs of controlled size for structural studies of membrane proteins by NMR. *Nat Protoc* **13**, 79–98 (2018).
